# Aging of immune system: Immune signature from peripheral blood lymphocyte subsets in 1068 healthy adults

**DOI:** 10.18632/aging.100894

**Published:** 2016-02-15

**Authors:** Ling Qin, Xie Jing, Zhifeng Qiu, Wei Cao, Yang Jiao, Jean-Pierre Routy, Taisheng Li

**Affiliations:** ^1^ Department of Infectious Diseases, Peking Union Medical College Hospital, Chinese Academy of Medical Sciences & Peking Union Medical College, Beijing, China; ^2^ Department of Internal Medicine, Peking Union Medical College Hospital, Chinese Academy of Medical Sciences & Peking Union Medical College, Beijing, China; ^3^ Chronic Viral Illnesses Service, McGill University Health Centre, Montreal, Quebec, Canada; ^4^ Research Institute of the McGill University Health Centre, Montreal, Quebec, Canada; ^5^ Division of Hematology, McGill University Health Centre, Montreal, Quebec, Canada

**Keywords:** lymphocyte subsets, aging, flow cytometry, reference range

## Abstract

Aging is a major risk factor for several conditions including neurodegenerative, cardiovascular diseases and cancer. Functional impairments in cellular pathways controlling genomic stability, and immune control have been identified. Biomarker of immune senescence is needed to improve vaccine response and to develop therapy to improve immune control. To identify phenotypic signature of circulating immune cells with aging, we enrolled 1068 Chinese healthy volunteers ranging from 18 to 80 years old. The decreased naïve CD4+ and CD8+ T cells, increased memory CD4+ or CD8+ T cells, loss of CD28 expression on T cells and reverse trend of CD38 and HLA-DR, were significant for aging of immune system. Conversely, the absolute counts and percentage of NK cells and CD19+B cells maintained stable in aging individuals. The Chinese reference ranges of absolute counts and percentage of peripheral lymphocyte in this study might be useful for future clinical evaluation.

## INTRODUCTION

The decline of the immune system named immunosenescence comprises a set of changes occurring to the innate and adaptive immune system that accompany human aging. These result in the increased susceptibility to infections, reduced effectiveness of vaccination and higher incidences of cancer, neurodegenerative disorders and metabolic imbalance in the elderly [1]. In order to meet the health related needs of the increasing elderly population, research on age-related immunosenescence needs to rapidly progress. Moreover, to develop appropriate preventative and therapeutic strategies, it is essential that we understand the underlying biological mechanisms that contribute to age-related pathogenesis. The aging process alters both the innate and the adaptive immune systems. Immunosenescence is characterized by a decrease in cell-mediated immune function as well as humoral immune responses. Age-dependent defects in T [2] and B cell [3] function coexist with age-related changes within the immune system. Two major features that lymphocytes acquire as they age are the loss of proliferative capacity [4] and the acquisition of typical markers of the NK cells [5]. In addition, a proportion of the elder population had a dramatic collapse in their B-cell repertoire diversity [6].

The immunophenotype alteration of lymphocytes subsets has been widely used in the evaluation of human immunodeficiency virus (HIV) infection [7], primary immunodeficiency [8], autoimmune diseases [9] and acute leukemia [10]. An examination of the phenotypic changes in circulating lymphocyte subsets is useful for monitoring the onset and progression of diseases and determining optimal treatment. Flow cytometric analysis is a convenient method for studying immune status and has been widely used for the clinical diagnosis and management of immune diseases associated with phenotypic and functional perturbations of lymphocyte subsets [11]. The studies on the immunophenotype of peripheral lymphocyte subsets in healthy people are limited. Infrequent sampling in healthy humans makes it difficult to capture an immune response in vivo. Some regional data of lymphocyte phenotypes show variations due to the influence of gender, age, ethnicity, and lifestyle differences [12]. These data indicate that different regional populations should have their own defined reference values for peripheral lymphocyte subsets. In order to better interpret the results of lymphocyte immuno-phenotyping in clinical practice, it is necessary to establish a reliable reference value of lymphocyte subsets in healthy people with different origins in various laboratory settings.

With the widely used flow cytometry, knowledge of dynamics of human lymphocyte subsets in peripheral blood during the long lifespan might provide an understanding of age-related immunosenescene. In this study, we aimed to display the age-related fluctuation of lymphocyte subsets in peripheral blood and partially explain the age-related lymphocyte senescence.

## RESULTS

### Reference range and variation for lymphocyte subsets in a long life span

A total of 1068 healthy Chinese adults were recruited for assessment of human immune cell compartmentalization, including 731 males (68.45%) and 337 females (31.55%). Their mean age was 40.5, 781 (73.13%) were in the young adult group (19-44 years old, 564 males, 217 females, mean age 35.4 years), 246 (23.03%) belonged to middle-aged adults (45-64 years old, 134 males,112 females, mean age 50.9 years) and 41 (3.84%) belonged to the elderly (65-80 years old, 33males, 8 females, mean age 71.6 years). The Chi-square test demonstrated that the gender was unbalanced among the three cohorts (*p*<0.001). The mean and 95% confidential interval (CI) of lymphocytes counts and percentage for each group ware shown in Table 1. Most parameters varied in different age group except for CD19+ B cell counts (*p*=0.383), CD19+ B percentage (*p*=0.863) CD3+CD4+ T cell counts (*p*=0.565, and CD4+CD28+ T cell counts (*p*=0.816) (Table 1). The absolute number and percentage of total (CD3+), helper (CD3+ CD4+), (CD3+ CD4+), cytotoxic (CD3+CD8+) T cells and natural killer (CD16+CD56+) cells in peripheral blood were outlined in Table 1. The absolute number and percentage of activated T cells (CD28+, CD38+ and HLA-DR) were also listed in Table 1.

**Table 1:** Reference range of T lymphocyte subsets in different age groups

parameters		All	Young	Middle-aged	Elder	*p* Value
		N=1068	N=781	N=246	N=41	
Age (years)	Mean ±SD	40.5±10.04	35.4±4.61	50.9±4.50	71.6±4.53	*-*
	Range	(19-80)	(19-44)	(45-64)	(65-80)	
Sex	Male : Female	731:337	564:217	134:112	33:8	0.000
Lymphocyte counts	Mean ±SD	2086 ±547	2106±535	2046±587	1946±505	0.042
(cells/ul)	95%CI	2053-2119	2068-2143	1972-2119	2053-2119	
CD19+ B counts	Mean ±SD	216 ±99	218±97	213±106	198±112	0.383
(cells/ul)	95%CI	210-222	211-225	200-226	163-233	
CD19+ B percentage (%)	Mean ±SD	10.40 ±3.73	10.38±3.56	10.49±4.02	10.17±5.00	0.863
	95%CI	10.18-10.63	10.14-10.64	9.9810.99	8.59-11.75	
CD16CD56+ NK counts	Mean ±SD	403 ±220	407±215	376±214	477±305	0.012
(cells/ul)	95%CI	389-416	392-422	349-403	381-574	
CD16CD56+ NK percentage	Mean ±SD	19.19 ±8.52	19.19±8.10	18.38±8.73	24.11±12.62	0.000
(%)	95%CI	18.68-19.70	18.62-19.75	17.29-19.48	20.13-28.09	
CD3+ T counts	Mean SD	1387 ±414	1403±402	1368±449	1198±399	0.006
(cells/ul)	95%CI	1362-1412	1375-1431	1312-1424	1071-1323	
CD3+ T percentage	Mean ±SD	66.44 ±8.58	66.62±8.17	66.67±8.96	61.73±12.12	0.002
(%)	95%CI	65.93-66.96	66.05-67.19	65.55-67.80	57.91-65.56	
CD3+CD4+T counts	Mean ±SD	694 ±202	690±227	708±241	699±281	0.565
(cells/ul)	95%CI	680-708	674-706	677-738	610-787	
CD3+CD4+/CD3+	Mean ±SD	33.48 ±7.33	32.91±6.95	34.89±7.80	35.81±9.80	0.000
(%)	95%CI	33.04-33.92	32.43-33.40	33.91-35.87	32.72-38.90	
CD3+CD8+T counts	Mean ±SD	589 ±244	605±234	561±269	448±235	0.000
(cells/ul)	95%CI	574-604	589-621	528-595	374-522	
CD3+CD8+/CD3+	Mean ±SD	27.96 ±7.67	28.52±7.27	26.97±8.12	23.09±9.91	0.000
(%)	95%CI	27.50-28.42	28.01-29.03	25.96-27.99	19.96-26.21	
CD4+CD45RA-	Mean ±SD	430 ±156	419±147	459±168	472±212	0.000
(cells/ul)	95%CI	421-440	408-429	438-480	406±539	
CD4+CD45RA-/CD4+	Mean ±SD	62.81 ±11.94	61.52±11.17	65.282±12.72	69.28±15.77	0.000
(%)	95%CI	62.09-63.52	60.73-62.30	64.23-67.42	64.30-7.25	
CD4+CD45RA+	Mean ±SD	264 ±138	271±133	249±147	226±165	0.000
(cells/ul)	95%CI	256-272	262-280	230-267	174-278	
CD4+CD45RA+/CD4+	Mean ±SD	37.19 ±11.94	38.48±11.17	34.18±12.72	30.73±15.78	0.017
(%)	95%CI	36.48-37.91	37.70-39.27	32.58-35.77	25.74-35.71	
CD4+CD45RA+CD62L+	Mean ±SD	240 ±129	247±125	221±132	206±160	0.004
(cells/ul)	95%CI	232-247	239-256	204-237	156-256	
CD4+CD45RA+CD62L+/CD4+	Mean ±SD	33.87±11.80	35.24±11.16	30.55±12.16	27.82±15.41	0.000
(%)	95%CI	33.17-34.58	34.45-36.02	29.03-32.08	22.95-32.68	
CD4+CD28+	Mean ±SD	617 ±210	619±206	616±212	597±271	0.816
(cells/ul)	95%CI	604-630	604-633	589-642	512-683	
CD4+CD28+/CD4+	Mean ±SD	89.18 ±8.44	89.88±7.47	87.56±10.01	85.59±12.52	0.000
(%)	95%CI	88.68-89.69	89.36-90.41	86.30-88.82	81.64-89.54	
CD8+CD28+	Mean ±SD	329 ±132	348±129	290±121	191±115	0.000
(cells/ul)	95%CI	321-337	339-357	274-305	155-228	
CD8+CD28+/CD8+	Mean ±SD	57.71 ±14.17	59.41±13.38	54.76±14.86	43.16±13.50	0.000
(%)	95%CI	56.86-58.56	58.47-60.35	52.89-56.62	38.90-47.42	
CD8+HLA-DR+	Mean ±SD	135 ±98	130±97	148±102	145±92	0.038
(cells/ul)	95%CI	129-141	124-137	135-161	116-174	
CD8+HLA-DR+/CD8+	Mean ±SD	22.38 ±11.08	20.78±10.55	25.77±11.18	32.59±10.34	0.000
(%)	95%CI	21.72-23.05	20.04-21.52	24.37-27.18	29.33-35.85	
CD8+CD38+	Mean ±SD	184 ±111	202±112	149±92	57±49	0.000
(cells/ul)	95%CI	178-191	194-210	138-161	42-73	
CD8+CD38+/CD8+	Mean ±SD	31.91 ±14.49	34.05±13.53	28.25±15.35	13.02±6.75	0.000
(%)	95%CI	31.04-32.78	33.10-35.00	26.32-30.18	10.89-15.15	
CD4+/CD8+	Mean ±SD	1.32 ±0.59	1.25±0.48	1.45±0.69	1.91±1.19	0.000
(%)	95%CI	1.29-1.36	1.22-1.29	1.37-1.54	1.54-2.29	

### CD16+CD56+ NK cell and CD19+ B cell keep balance with older ages

NK cell counts in peripheral blood of the elderly seemed slightly higher than that of younger people in Table 1. However, further correlation analysis found that age did not influence NK cells (counts: *r*=0.041 *p*=0.18; per-centage=0.001 *p*=0.977) or B cells (counts: *r*=−0.041, *p*=0.181; percentage=−0.005 *p*=0.86) significantly in our study.

### CD3+ T cell counts, CD3+CD8+ T cell counts, CD4+ CD45RA+ T cell counts, CD4+ CD45RA+CD45SRA+ CD62L+ T cell counts, CD4+CD28+ /CD4+ ratio and CD8+CD28+ /CD8+ ratio decrease with older ages

**Figure 1:**
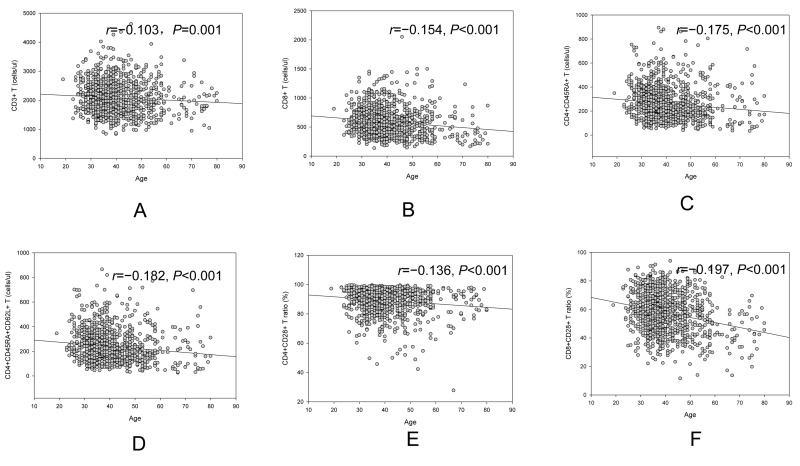
Age related change of CD3+ cell (**A**), CD3+CD8+ (**B**), CD4+CD45RA+ cell (**C**), CD4+CD45RA+CD62L+ cell (**D**), CD4+CD28+ /CD4+ (**E**) and CD8+CD28+/CD8+ (**F**).

A trend of decrease in CD3+ T cell counts (*r*=−0.103, *p*=0.001, Figure 1A), CD3+CD8+ T cell counts (*r*=−0.154, *p*<0.001, Figure 1B), CD4+CD45RA+ T cell counts(*r*=−0.175, *p*<0.001, Figure 1C), CD4+ CD45RA+CD62L+ T cell counts(*r*=−0.182, *p*<0.001, Figure 1D), CD4+CD28+ /CD4+ ratio (*r*=−0.136, *p*<0.001, Figure 1E) and CD8+CD28+/CD8+ ratio (*r*=−0.197, *p*<0.001, Figure 1F) was observed with increased age. An increase trend with aging in CD4+/CD8+ ratio (*r*=0.160, *p*<0.001) was showed in our study.

### A reverse age related trend in CD4+CD45RA+ T cell counts and CD4+CD45RA− T cell counts, CD8+HLA-DR/CD8+ ratio and CD8+CD38+/CD8+ ratio

**Figure 2:**
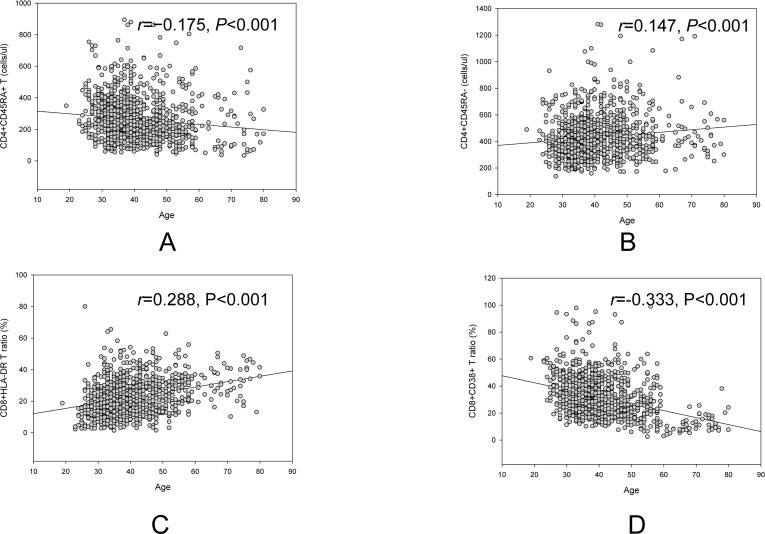
Reverse age related trend of naive CD4+ (**A**) and memory CD4+ (**B**), CD8+HLA-DR ratio (**C**) and CD8+CD38+ ratio (**D**).

Overall, there was a decrease age related trend of CD4+CD45RA+ T cell counts (*r*=−0.175, *p*<0.001, Figure 2A), but an increase age related trend of CD4+CD45RA− T cell counts (*r*=0.147, *p*<0.001, Figure 2B). Another reverse trend for CD8+HLA-DR/CD8+ ratio (*r*=0.288, *p*<0.001, Figure 2C) and CD8+CD38+/CD8+ ratio (*r*=−0.333, *p*<0.001, Figure 2D) was also showed in figure 2.

### Age related changes by every 10 years for lymphocyte subsets

**Figure 3:**
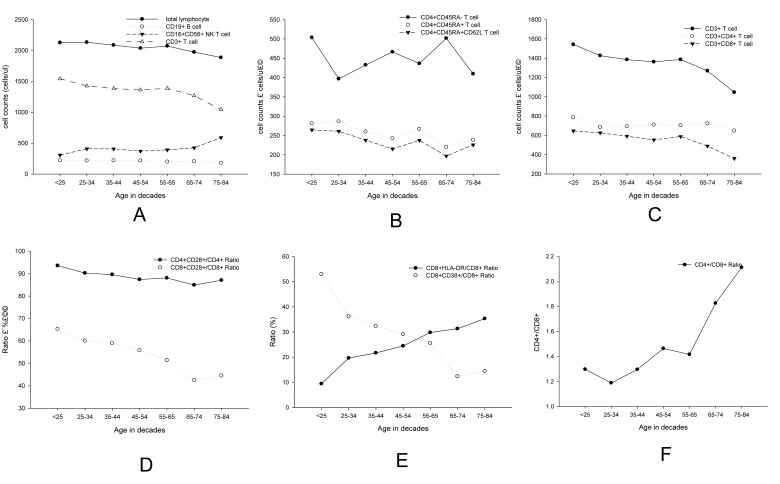
Linear diagrams showed age-related changes by decade for lymphocyte subsets of (**A**) total lymphocyte cells, CD19+ (**B**) cell, CD16CD56+ NK cell and CD3+ T cell B CD3+ T cell, CD4+ T cell and CD8+ T cell; (**C**) CD4+CD45RA−, CD4+CD45RA+ and D4+CD45RA+CD62L+ (**D**) CD4+CD28+, CD8+CD28+ (**E**) CD8+HLA.DR+/CD8+ and CD8+CD38+/CD8+ (**F**) CD4+/CD8+.

For further comparisons, we divided the subjects by every 10 years, as shown in Table 2. We further observed the age-related changes by every 10 years for lymphocyte subsets (Figure 3). Although similar trends were obtained for all parameters, fluctuation could be observed in different parts of the curves.

**Table 2:** Age distribution by every decade

	Younger than 25	25—34	35—44	45—54	55—64	65—74	75 and above
number	7	312	462	186	60	28	13
% in whole	0.65	29.21	43.26	17.42	5.62	2.62	1.22

### Gender influence on the fluctuation of lymphocyte subsets

As the gender was unbalanced among the three cohorts, we compared the counts and percentage of lymphocyte subsets. Most parameters were comparable between genders, except for the NK cells, B cells, CD3+T cells, CD3+CD8+ T cells, CD8+CD28+ T cells, CD8+HLA-DR and CD4+/CD8+ ratio (Table 3). The analysis showed that certain T cell subsets were affected by gender. Therefore, we use multivariate logistic regression to assess the age related variation for lympho-cytes subsets (Table 4). Age did not seem to influence CD4+ T cell counts, B cell counts and percentage, NK cell counts and percentage, and CD4+CD28+ T cell counts significantly in our study. However, for the majority of the parameters, such as CD4+/CD8+ ratio, the percentage of CD3+CD4+ T cells and CD4+CD28+ T cells, the absolute counts and percentage of CD3+T cells, CD3+CD8+ T cells, CD4+CD45RA+ T cells, CD45+CDRA− T cells, CD45+CD45RA+CD62L+ T cells, CD8+CD28+ T cells, CD8+HLA-DR and CD8+CD38+, significant relation with aging were observed in Table 4.

**Table 3:** Reference range of T lymphocyte subsets in different gender

	Male		Female		
Parameters	Mean ±SD	95%CI	Mean ±SD	95%CI	*p* value(M&F)
CD19+ B counts (cells/ul)	223.5±105.40	129.95-315.75	215.7±98.45	121.31-276.50	0.001
CD19+ B percentage (%)	10.2±3.63	6.55-13.84	10.3±3.59	7.13-14.20	0.031
CD16+CD56+ NK counts(cells/ul)	448.0±223.25	244.49-650.87	407.5±216.91	160.72-470.68	<0.001
CD16+CD56+ NK percentage (%)	20.4±8.33	12.20-28.81	19.3±8.21	9.62-23.73	<0.001
CD3+ T counts (cells/ul)	1438.6±418.98	1031.80-1845.75	1394.0±417.15	916.46-1672.80	<0.001
CD3+ T percentage (%)	65.4±8.57	56.55-74.40	66.4±8.39	60.62-76.35	<0.001
CD3+CD4+ counts (cells/ul)	696.0±232.80	478.02-912.79	688.2±228.78	466.85-874.61	0.139
CD3+CD4+/CD3+ (%)	31.8±6.83	24.88-38.74	33.0±6.94	29.21-42.21	<0.001
CD3+CD8+ counts (cells/ul)	631.1±254.60	389.50-873.19	597.5±246.38	328.11-716.92	<0.001
CD3+CD8+/CD3+ (%)	28.5±7.92	20.64-36.40	28.2±7.60	20.70-34.31	0.125
CD4+CD45RA− (cells/ul)	429.0±156.34	286.61-570.10	424.6±150.57	289.36-541.15	0.347
CD4+CD45RA−/CD4+ (%)	62.3±11.53	50.07-74.79	62.5±11.50	51.16-74.96	0.390
CD4+CD45RA+ (cells/ul)	267.0±133.68	142.48-391.46	263.6±134.54	133.24-377.01	0.143
CD4+CD45RA+/CD4+ (%)	37.7±11.53	25.61-49.91	37.5±11.50	25.04-49.13	0.413
CD4+CD45RA+CD62L+ (cells/ul)	242.8±124.86	126.60-359.19	240.0±126.47	117.93-347.58	0.160
CD4+CD45RA+CD62L+/CD4+ (%)	34.4±11.48	22.26-46.82	34.2±11.45	21.95-45.72	0.470
CD4+CD28+ (cells/ul)	616.5±204.90	423.33-808.88	611.5±205.14	410.02-790.52	0.228
CD4+CD28+/CD4+ (%)	89.1±8.38	79.76-98.40	89.2±8.16	80.89-98.14	0.425
CD8+CD28+ (cells/ul)	349.9±132.85	221.31-479.19	336.0±130.06	189.24-422.56	<0.001
CD8+CD28+/CD8+ (%)	57.7±14.01	43.00-72.51	58.4±13.80	45.18-75.25	0.017
CD8+HLA-DR (cells/ul)	147.3±102.74	58.20-236.70	138.0±99.20	47.78-184.00	<0.001
CD8+HLA-DR/CD8+ (%)	22.7±10.74	11.94-33.92	22.4±10.72	11.65-32.10	0.267
CD8+CD38+ (cells/ul)	191.6±111.9	99.28-280.28	190.0±108.43	95.84-277.46	0.535
CD8+CD38+/CD8+ (%)	31.2±13.16	18.91-43.27	32.7±13.74	22.61-50.00	0.552
CD4+/CD8+ ratio (%)	1.2±0.54	0.75-1.71	1.3±0.54	0.93-1.87	<0.001

**Table 4:** Relationship between age and T lymphocyte subsets in regression analysis

Age & T lymphocyte subsets	Unstandardized coefficient	Standard Error	*p* value
CD19+ B counts	−6.129	5.667	0.280
CD19+ B percentage	−0.409	0.212	0.818
CD16CD56+ NK counts	8.708	12.083	0.471
CD16CD56+ NK percentage	0.987	0.474	0.057
CD3+ T counts	−56.889	23.432	0.015
CD3+ T percentage	−1.329	0.482	0.006
CD3+CD4+T counts	12.45	13.28	0.349
CD3+CD4+T percentage	1.427	0.402	0.000
CD3+CD8+T counts	−52.044	13.611	0.000
CD3+CD8+T percentage	−2.010	0.433	0.000
CD4+CD45RA-	34.761	8.856	0.000
CD4+CD45RA− percentage	4.12	0.67	0.000
CD4+CD45RA+	−22.304	7.841	0.005
CD4+CD45RA+ percentage	−4.119	0.670	0.000
CD4+CD45RA+CD62L+	−23.870	7.391	0.001
CD4+CD45RA+CD62L+ percentage	−4.239	0.662	0.000
CD4+CD28+	−6.776	12.015	0.573
CD4+CD28+/CD4+	−2.333	0.476	0.000
CD8+CD28+	−65.54	7.214	0.000
CD8+CD28+/CD8+	−6.501	0.781	0.000
CD8+HLA-DR+	15.939	5.522	0.004
CD8+HLA-DR+/CD8+	5.625	0.606	0.000
CD8+CD38+	−62.639	6.036	0.000
CD8+CD38+/CD8+	−8.518	0.765	0.000
CD4+/CD8+	0.248	0.033	0.000

## DISCUSSION

The aging process seems to alter both branches of the immune system, the innate and the adaptive, in different ways. While the adaptive immune response undergoes profound age-dependent modifications [3], innate immunity has been considered to be better preserved globally [5]. In this study, we showed series of changes in lymphocyte subsets that accompany human aging.

### CD3+ T lymphocytes decrease with aging

Age-related decrease occurred in CD3+ T cell and CD3+CD8+ T cell count, but not in CD3+CD4+ T cell count in our study. Although the CD3+CD4+ T cell count had maintained balance in different age subgroup, but age-related reverse trends of naïve CD4+ T and memory CD4+ T lymphocyte were also clearly displayed. In our analysis, CD4+CD45RA+ CD62L+ T cell, a naïve CD4+ T cell subset, showed age-related decrease. The thymus is a primary lymphoid organ that plays a crucial role in the development of T lymphocytes by providing a suitable microenvironment where these cells can proliferate, rearranging the T cell receptor (TCR) and maturate to mount an adequate immune response against foreign pathogens and tumor cells. Throughout the lifetime it provides a continuous supply of naive T cells, though shortly after the start of youth, a reduction in the overall thymic size and a replacement of the functional tissue by fat begin to take place, resulting in increasingly fewer naive T cells exit to the periphery [13].

Thymic involution may represent a mechanism of maintaining a sufficiently diverse repertoire to combat a variety of potential pathogens and avoid autoimmune reactions. The possible reasons of thymus involution may be the blocking of TCR gene rearrangement, self-peptide MHC decreased molecules [14], and loss of T cell progenitors [15]. The dynamic processes of repeated interaction with cognate antigens lead to multiple division cycles involving a high degree of cell differentiation, senescence, restriction of the TCR repertoire, and cell cycle arrest [16]. This cell cycle arrest is associated with the loss of telomere sequences from the ends of chromosomes. Telomere length is reduced at each cell cycle, and critically short telomeres recruit components of the DNA repair machinery and trigger replicative senescence or apoptosis [17]. Stimulated T cells become refractory to telomerase induction, suffer from telomere erosion and enter into replicative senescence [18]. As a consequence, the number of CD3+ T cells, especially naive T cells, exiting the thymus is dramatically decreased with aging.

Both naïve CD4+ and CD8+ T lymphocytes arise from the thymus, but the behavior of naive T cells is different in CD4+ and CD8+ compartments, where naive CD4+ T cells modestly decline with age, while naive CD8+ T cells plummet at the earlier stage of life[19]. This indicated that in the absence of significant thymic influx, the rolling over of naive CD4+ T cells is sufficiently sustained for another two to three decades by homeostatic proliferation. The mechanism of this rapid decline in naïve CD8+ T lymphocytes remains unknown. Several studies have proposed that different growth factors and cytokines may be involved in regulating the two distinct populations of lymphocytes [20]. Senescence of the naïve CD8+ subpopulation take place even earlier in life, which may explain the fact that age-related decrease of CD3+CD8+ T cells occurs earlier than that of CD3+CD4+ T cells.

### CD28- T lymphocytes associated with age related functional immune response

In this study, we examined the effect of age on CD28 expression in CD4+ and CD8+T cells. The CD4+CD28-T cells are less frequent than CD8+CD28- T cells in elder people. As humans age and consequently augment their antigen experience, they accumulate CD28- T cells, mostly within the CD8 subset. This is presumably driven by the cumulative exposure to persistent antigens. CD8+T cells play a central role in the recognition and clearance of intracellular pathogens.

Upon an initial antigen exposure, CD28 clearly helps to ensure that CD8+ T cell responses are initiated solely when antigen is presented by an antigen presenting cell that has been activated. Properly presented antigen elicits an appropriate immune response culminating in the retention of a small population of CD8+ memory T cells. Survival after an antigen exposure suggests that the immune response was indeed appropriate, and the requirement for co-stimulation might be considered an unnecessary redundancy delaying a protective response. Therefore, memory CD8+ T cells are generated and maintained for defense against subsequent exposures to the same antigens, enabling a faster and vigorous response. Repeated antigen stimulations induce progressive reduction in CD28 expression on the surface of CD8+T cells, eventually generating a population of highly antigen experienced CD8+CD28-T cells [21]. Furthermore, age-related thymic involution and its related reduced output of naïve CD28+ T cells may also contribute to an aged and weakened immune phenotype. At birth, virtually all human T cells express CD28. In young adults, up to 20–30% of their CD8+ T lymphocytes lose CD28 expression. In individuals over 80 years old, over 50–60% of their CD8+ T cells lose CD28 expression [22]. The detailed mechanism is unclear and it is thought that common chronic viral infections including human cytomegalovirus (CMV) and Epstein–Barr virus (EBV) contribute to the CD8+CD28- T cell population expansions [23]. Two longitudinal studies in Swedish octogenarians and nonagenarians cohorts studies [24] showed the identification of the immune biomarkers associated with increased mortality, named immune risk phenotype (IRP). High mortality has been found in healthy elderly individuals with an IRP, featured by the inverse of CD4/CD8 ratio (less than 1), loss of naïve T cells, poor T-cell proliferative responses to mitogens, increased CD8+CD28-CD57+ cell, low number of B cells and clonal expansions of CMV or EBV specific CD8 T cells.

Molecular CD28 represents a very important co-stimulatory marker for effector CD4+ and CD8+ T lymphocyte. Loss of CD28 has been reported as a key predictor of immune incompetence in elderly people. CD28- T cells have decreased antigen receptor diversity, compromised antigen-induced proliferation, and are limited by a shorter replicative lifespan, though they exhibit enhanced cytotoxic and regulatory functions. These characteristics may contribute to the immune incompetence in the elderly, as manifested by susceptibility to latent viral reactivation, and compromised responses to novel pathogens, cancer cells and vaccines [25]. Therefore, CD28 loss has been associated with physiologic degeneration and poor response to vaccine in humans. Normal aging is directly correlated with the oligoclonal accumulation of CD8+ CD28- T cells. CD28- T cells may just be residual cells from prior antigen exposures, and the interplay of costimulatory and coinhibitory pathways in the context of CD28 loss will play an immense role in therapeutic development for many human diseases and immunsenescence.

### Variable CD8+ T cell activation markers accompanied with aging

A significant increase of CD8+HLA-DR/CD8+ and a decrease trend of CD8+ CD38+ /CD8+ with aging were noted in our analysis, in accordance with previous report [26]. CD38 was identified in the late 1970s, and involved in antigen recognition. CD38 was initially found on thymocytes and T lymphocytes, later the molecule was found throughout the immune system, although its expression levels vary. Because of this, CD38 was considered an ‘activation marker’ a term still popular in routine flow cytometry. CD38 plays dual roles as receptors and ectoenzymes, endowed with complex activities related to signaling and cell homeostasis. An initial function attributed to CD38 was the regulation of activation and proliferation of human T lymphocytes. Otherwise, CD38 is a multifunctional enzyme that catalyzes the synthesis of cyclic ADP ribose (cADPR) which is involved in regulation of cytoplasmic Ca^2+^ influxes, activating signaling pathways critical for several biological processes [27]. High ratios of CD38+/CD8+T lymphocytes predict disease progression and strong immunosuppressive status in HIV-infected adults [28]. Immune senescence in HIV infection patients is clinically characterized by increased expression of CD38, apparently not directly caused by the infection [29]. CD38 expression has been also matter of technological debate, in terms of monoclonal antibody used in HIV patients. CD38 is also reported as target of autoantibodies in diabetes mellitus [30] and SLE.

The knowledge of CD8+CD38+ or CD8+HLA-DR role is still very poor in the aging process and most studies were conducted in HIV infected individuals. High proportions of CD38+CD8+ T cells were considered as a marker of poor response to therapy and prognosis in AIDS [31]. As a marker of T cell activation, CD38+ expression on T cells could predict acute graft versus host disease [32]. However, the mechanism of age related changes of T activation marker, CD38 or HLA-DR, still remains unclear.

### NK cells and B cells

The age-related increase of NK cells is well documented and the detailed cytofluorimetric analysis allowed us to demonstrate an age-related increase of cells with high NK activity [33]. However, we did not observe such the above mentioned phenomenon or any relationship between age and NK cells or B cells.

Age has been reported to be associated with changes in the numbers, phenotype and function of NK cells. In healthy elderly individuals, the age-associated increasing of NK cell counts and remodeling of NK cell subsets has been described characterized by a decreased percentage of the more immature CD56bright NK cells and an increase of CD56− NK cells[34]. B cell is always considered as antibody producers, but they are also highly effective as antigen presenting cells, and essential for the development of memory T-cell. Although B cell counts were not significantly changed during long lifetime, the collapse in B cell diversity had been found easily [35]. Some very old individuals show a dramatic reduction in B cell diversity which is linked to frailty. The collapse of diversity is a strongly predictor of poor health status in elderly people [36].

### The variation of lymphocyte subsets counts in different area

The reference values of the main circulating lymphocyte subsets have been established by many studies throughout the world, and have shown some variability according to specific locations (Table 5). The data of lymphocyte subsets in Chinese adults are different from those of people in other areas of the world, probably due to difference in races, antigen exposure and living environments. We closely apply flow cytometry analysis techniques to describe lymphocyte repertoires in a group (>1000 persons) of Chinese adults with wide age range. The valid comparison of lymphocyte subsets analysis, using the same flow cytometry methods, should be made between our data and other countries' data in the population with similar age reference range and sex ratio (Table 5). It seems that the genetic, nutrition and environmental variations between populations in different area might be possible causes of the differences in lymphocyte subsets. Our results suggested that region reference ranges for lymphocyte subsets were necessary.

**Table 5:** comparison lymphocyte subsets of present study with other studies

Area	country	Year	Num	Age range	Male: Female	CD3+ CD4+	CD3+ CD8+	CD4+/CD8+ ratio	CD16+CD56+	CD19+
				(years)		(cells/ul)	(cells/ul)		(cells /ul)	(cells /ul)
Asia	China	Present	1068	19-80	731:337	694	589	1.32	403	216
	motherland	study								
	Singapore	2004	232	10-69	104:128	838	642	1.43	419	353
	Iran	2011	233	20-45	150:83	827	522	1.61	248	332
	HongKong	2013	273	17-59	150:123	760	515	1.59	229	298
	Korea	2014	294	33-61	139:155	787	479	1.81	300	203
Africa	Ethiopia	1999	485	15-45	280:205	775	747	1.2	250	191
	Nigeria	2009	2570	18-80	1363:1207	847	435	2.3	-	-
	Morocoan	2012	242	19-49	220:22	871	637	1.37	211	59
	Kenyan	2013	315	16-60	222:85	920	646	1.57	-	-
America	Mexico	2013	400	20-40	200:200	818	528	1.5	-	-
	Florida	2014	50	31-67	25:25	1003	590	1.8	214	256
	Brazil	2015	238	16-56	134:104	844	555	1.52	234	252
Europe	Italy	1999	968	18-70	532:436	940	551	1.71	278	230
	Switzerland	2004	70	24-70	44:26	691	343	2.1	184	170
	German	2005	100	19-85	50:50	870	460	1.9	280	220

### Gender-related difference in immune cell numbers or percentage

In our study, the gender influence on lymphocyte subsets was clear shown. Previous studies also demonstrated the gender-related difference in immune cell numbers or percentage [37]. The differential influences of sex hormones could explain that phenomenon. Androgens accelerate thymocyte apoptosis and may in turn shape the peripheral T cell repertoire [38].

## MATERIALS AND METHODS

### Subjects

All the subjects were healthy volunteers recruited between February 2007 and July 2011, with ages from 18 to 80 years old. The recruitment was strictly conducted according to the defined criteria from the SENIEUR protocol guideline [39, 40]. Subjects testing positive to HIV, systemic infection, connective tissue disease, abnormal tumor marker or cancer were excluded. Informed consent was obtained from all subjects, and the Ethical Committee of Peking Union Medical College Hospital approved this study. According to the definition by the National Health and Family Planning Commission of the People's Republic of China, the subjects were classified as elderly (≥65), middle-aged (45-64), and young (18-44) adults.

### Lymphocyte immunophenotyping

Immunophenotyping of peripherial blood lymphocytes was analyzed by three-color flow cytometry (Epics XL flow cytometry; Bechman Coulter, USA) as previously described [41-42]. Freshly collected EDTA-anticoagulated whole blood was incubated and tested with a panel of monoclonal antibodies directed against fluorescein isothiocyanate/phycoerythrin/peridinin chlorophyll protein combinations of CD3/CD8/CD4, CD3/CD16CD56/CD19, HLA-DR/CD38/CD8, CD28/CD8/CD4, CD62L/CD45RA/CD4 and isotype controls (Immunotech, France). Cell counts of lymphocyte subsets were calculated using a dual-platform method with the white blood cell counts and lymphocyte differentials obtained from blood routine tests of the same specimen.

### Statistical analysis

Statistical analysis was performed using SPSS software (SPSS® for Windows™ version 13.0, SPSS Inc., Chicago, IL, USA). Kolmogorov-Smirnov was used for the distribution test. Reference ranges were calculated using mean ± 2 standard deviation for parametric data and 2.5% and 97.5% percentiles for non-parametric data. Comparisons among three variables were performed using one-way analysis of variance. Genders were compared using t-test for parametric data and Mann–Whitney test for non-parametric data. As gender was a significant determinant of fluctuation of T lymphocyte subset, multiple linear logistic regression was used for analyzing the relation between aging and T lymphocyte subsets. Association between variables and age was tested using a non-parametric Spearman's rank correlation test. Probability value was obtained from 2-sided tests and P<0.05 was considered statistically significant.

## Conclusion

Immunosenescence comprises a set of changes occurring to the peripheral T lymphocyte subsets. In this study, we used flow cytometric immuno-phenotyping to evaluate the counts and percentage of circulating lymphocyte subsets in health young and older adults. Several fluctuations of T lymphocyte subsets accompanied with aging, including decreased naïve CD4+ or CD8+ T cells, increased memory CD4+ or CD8+ T cells, loss CD28 expression on T cell and reverse change trend of CD38+ and HLA-DR, might show clues for immunosenescence of immune system in the further study. Besides that, the establishment of reference ranges for peripheral blood lympho-cyte subsets in healthy adults of different age group might be used to guide clinical evaluation.

## Limitation

Firstly, our data had only enrolled lymphocyte subsets of peripheral blood, which contained less than 3% of the total T cells in the body, without analysis that of tissue, such as spleen, lymph node or interstitial muco-sa. Secondly, the percentage of healthy older people enrolled in this study is limit. However, the reliable mapping of human T cells in different decades was reliable in our cohort and the trend of a set of T cell changes accompanied with aging was primarily displayed.
